# Digital health technology to support care and improve outcomes of chronic kidney disease patients: as a case illustration, the Withings toolkit health sensing tools

**DOI:** 10.3389/fneph.2023.1148565

**Published:** 2023-04-24

**Authors:** Bernard Canaud, Jeroen Kooman, Andrew Davenport, David Campo, Eric Carreel, Marion Morena-Carrere, Jean-Paul Cristol

**Affiliations:** ^1^ Montpellier University, School of Medicine, Montpellier, France; ^2^ Global Medical Office, Fresenius Medical Care (FMC), Fresnes, France; ^3^ Department of Internal Medicine, Division of Nephrology, Maastricht University Medical Center, Maastricht, Netherlands; ^4^ UCL Department of Renal Medicine, Royal Free Hospital, University College, London, United Kingdom; ^5^ Withings, Issy les Molineaux, France; ^6^ PhyMedExp, University of Montpellier, INSERM, CNRS, Department of Biochemistry and Hormonology, University Hospital Center of Montpellier, Montpellier, France; ^7^ AIDER-Santé, Ch. Mion Foundation, Montpellier, France

**Keywords:** chronic kidney disease, hemodialysis, pervasive remote monitoring, digital connected health, CKD, empowering patient

## Abstract

Cardiovascular disease (CVD) is a major burden in dialysis-dependent chronic kidney disease (CKD5D) patients. Several factors contribute to this vulnerability including traditional risk factors such as age, gender, life style and comorbidities, and non-traditional ones as part of dialysis-induced systemic stress. In this context, it appears of utmost importance to bring a closer attention to CVD monitoring in caring for CKD5D patients to ensure early and appropriate intervention for improving their outcomes. Interestingly, new home-used, self-operated, connected medical devices offer convenient and new tools for monitoring in a fully automated and ambulatory mode CKD5D patients during the interdialytic period. Sensoring devices are installed with WiFi or Bluetooth. Some devices are also available in a cellular version such as the Withings Remote Patient Monitoring (RPM) solution. These devices analyze the data and upload the results to Withings HDS (Hybrid data security) platform servers. Data visualization can be viewed by the patient using the Withings Health Mate application on a smartphone, or with a web interface. Health Care Professionals (HCP) can also visualize patient data *via* the Withings web-based RPM interface. In this narrative essay, we analyze the clinical potential of pervasive wearable sensors for monitoring ambulatory dialysis patients and provide an assessment of such toolkit digital medical health devices currently available on the market. These devices offer a fully automated, unobtrusive and remote monitoring of main vital functions in ambulatory subjects. These unique features provide a multidimensional assessment of ambulatory CKD5D patients covering most physiologic functionalities, detecting unexpected disorders (i.e., volume overload, arrhythmias, sleep disorders) and allowing physicians to judge patient’s response to treatment and recommendations. In the future, the wider availability of such pervasive health sensing and digital technology to monitor patients at an affordable cost price will improve the personalized management of CKD5D patients, so potentially resulting in improvements in patient quality of life and survival.

## Introduction

Cardiovascular disease (CVD) is a major burden in dialysis-dependent chronic kidney disease (CKD5D) patients (1). Mortality from cardiovascular disease is 10 to 20 times higher in CKD5D patients than in the general population matched for age and gender (2). The pathophysiologic link between CVD and CKD is well-established involving multiple factors including dialysis prescription and monitoring (3). Severe CVD events are the main cause of death in CKD5D patients (4). Deaths from cardiovascular disease accounted for 48% of all deaths, including arrhythmias, cardiac arrest, congestive heart failure (CHF), acute myocardial infarction (AMI), and atherosclerotic heart disease (ASHD) in the annual USRDS 2019 report (5). Interestingly, arrhythmias and cardiac arrest represented 50% of all cardiac deaths.

**Figure 1:**
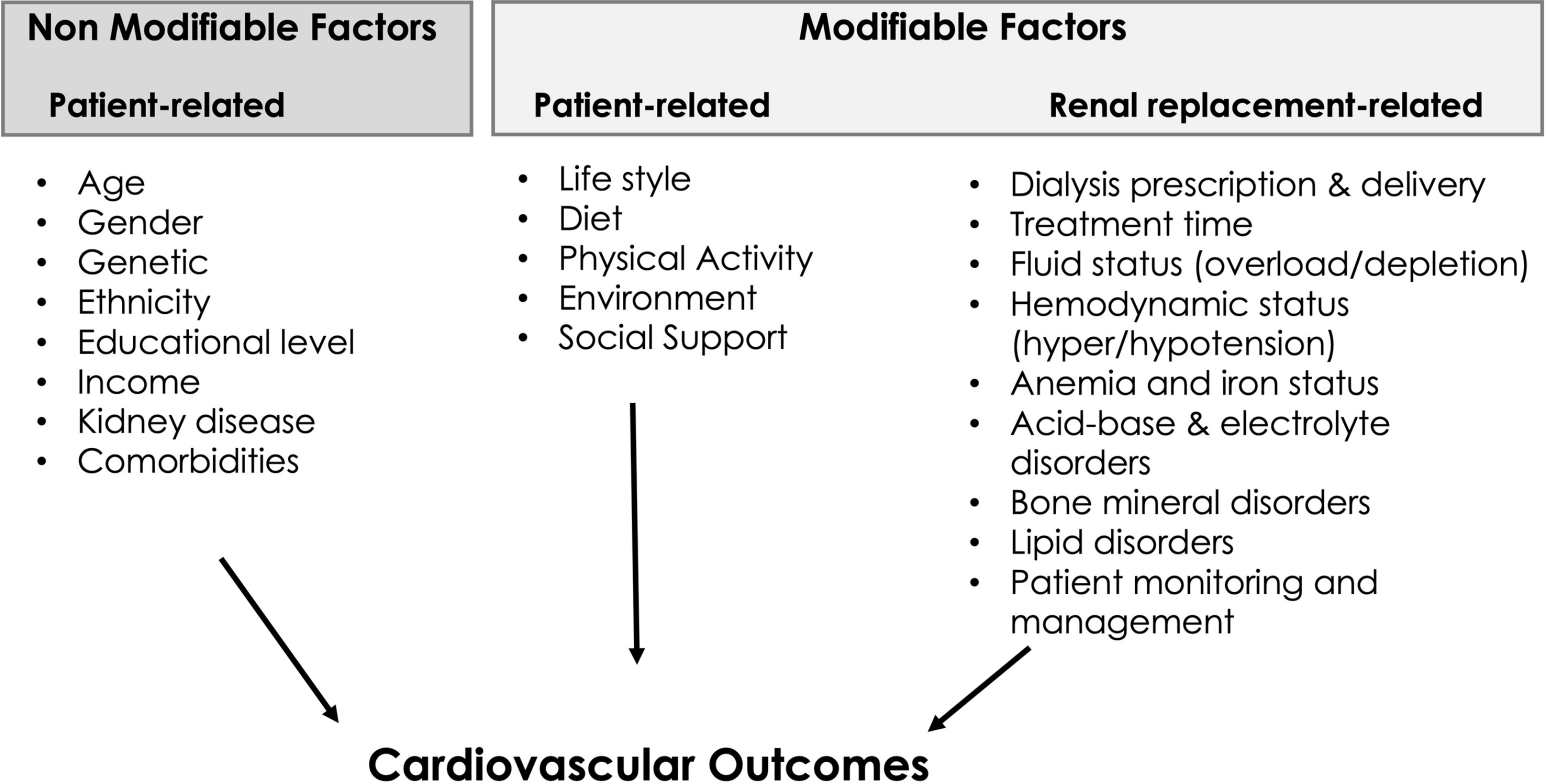
Factors affecting cardiovascular outcomes in CKD5D patients.

It is widely recognized that chronic kidney disease patients represent a high cardiovascular risk population (1, 6). Several factors contribute to this vulnerability including traditional ones namely age, gender, life-style and comorbidities, and non-traditional ones such as nephropathy, volume overload, hypertension, uremic and metabolic disorders, uremic cardiomyopathy and renal replacement modality (7). These are summarized in **Figure 1**. It is not our intention to review all these factors, but rather to highlight the fact that CKD5D patient management and dialysis modality are modifiable factors that may have a strong influence on patient outcomes (8).

By triggering cardiac events, conventional thrice weekly hemodialysis is a significant potential disease modifier as indicated by recent studies. Large cohort studies have shown that interdialytic periods, in particular those with the longest interval (2 days break), increased the risk for hospitalization and mortality by 20 to 40% (9–11). Importantly, these risks are much greater in the 12 hours following the hemodialysis session (early) and in the 12 hours preceding the next hemodialysis session (late). These findings suggest that two different mechanisms are likely involved: the early one would favor a role for rapid fluid and electrolyte shifts along with changes in cardiac structure and perfusion and sympathetic-vagal nervous system imbalance (12–14); the late one would favor a role for fluid overload, hypertension and hyperkalemia (15–17). Recent prospective studies using either skin surface patch or implantable loop recorders allowing continuous electrocardiogram (ECG) recording brought new insights into this problem (18). Based on ambulatory ECG monitoring, it was shown that clinically significant arrhythmias were quite common and more frequent in hemodialysis patients than expected (19, 20). It is interesting noting that among clinically symptomatic arrhythmias, bradycardias, which sometimes were a harbinger of asystole, were more frequently observed than ventricular tachycardia and also that these rhythmic disorders were strongly related to the dialysis cycle. Next to this, studies with implantable loop recorders also showed that atrial fibrillation was prevalent in nearly 30% of dialysis patients, and episodes of atrial fibrillation (AF) related to the dialysis cycle. Although the optimal treatment strategy for AF in patients on HD remains under discussion, these findings are of relevance, as AF in patients with CKD 5D is strongly associated with mortality.

In this context, it appears of utmost importance to bring a closer attention to CVD monitoring in caring for CKD5D patients to ensure early and appropriate interventions to improve their outcomes (21). Technological advances had led to the development of new home-used, self-operated, connected medical devices, which offer convenient and new tools for monitoring patients in a fully automated and ambulatory mode during the interdialytic period for CKD5D patients.

In this narrative essay, we discuss the clinical potential of pervasive wearable sensors for monitoring ambulatory dialysis patients. On purpose, we selected Withings’ as specific digital health care equipment for our case illustration. We recognized that several digital remote monitoring equipment are available on the market (i.e., Apple, Samsung, Gyant, Medopad, Chronisense Medical, iHealth, Vitls) that have their own unique features and specific applications. To our knowledge however, these companies don’t provide yet a comprehensive and combined approach of monitoring chronic ambulatory patients in their various vital dimensions (i.e., weight, blood pressure, fluid status, heart rhythm, sleep disorders). In addition, these companies are not capturing, analyzing data in a centralized cloud-based system, able to empower patients or support healthcare professionals in their decision making. Withings’ provides today a toolkit digital medical health device with remote patient monitoring system, cloud-based advanced analytic system, supported by clinical validation that may be easily implemented in daily practice.

## Digital health tools: the example of Withings as a multiplexed integrated web-based architecture system

### Analytic description: pervasive and non-intrusive connected tools

**Figure 2:**
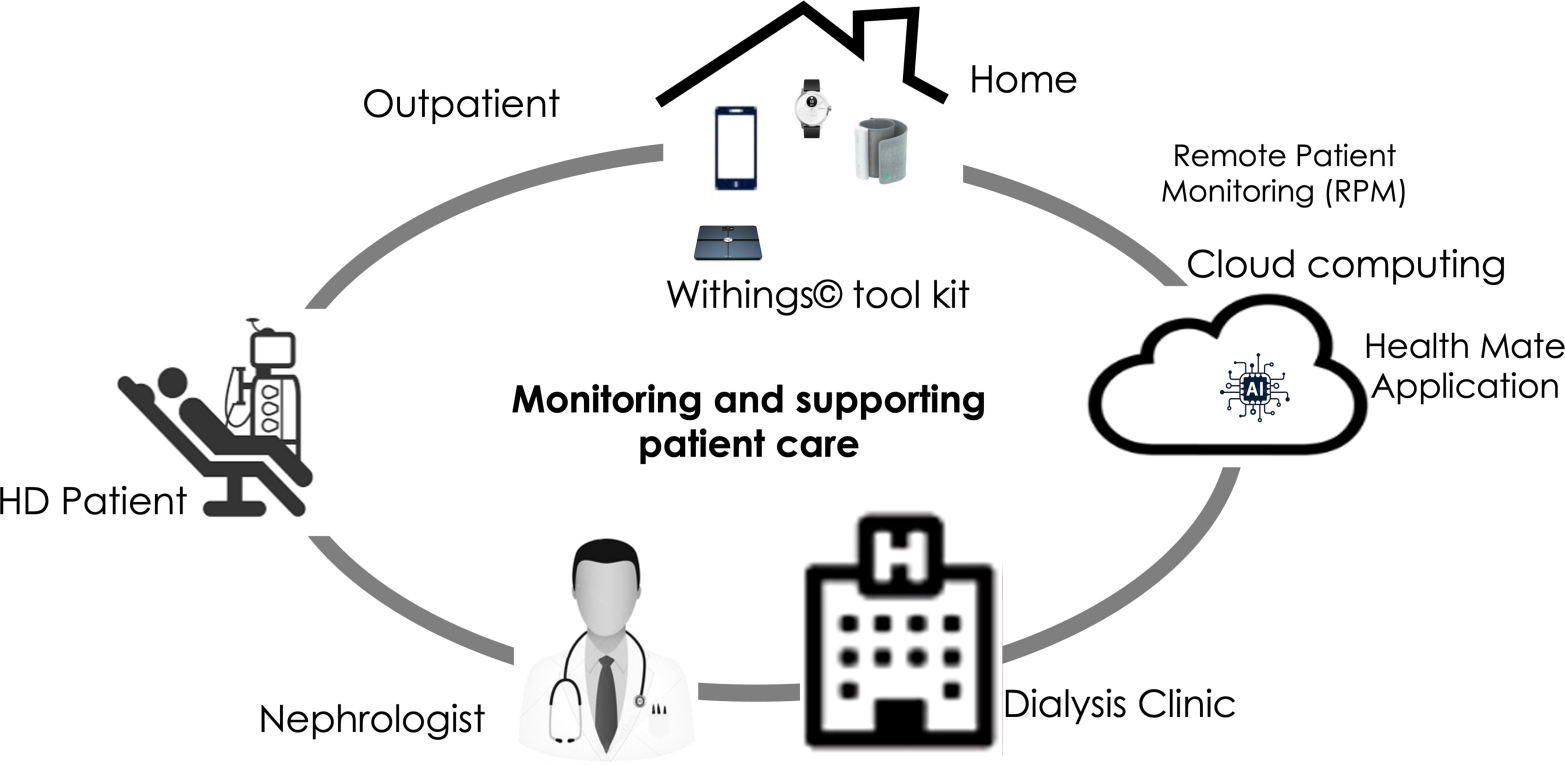
Digital health tools to support and improve management of dialysis patients.

Withings, an electronics company has pioneered several devices aimed at facilitating the remote monitoring of chronically ill patients. Withings tool kit digital devices and their potential use in dialysis patients are presented in **Figure 2**.

Scanwatch^©^ is an analog watch, which can allow the patient to record a 30 second single-lead ECG and make spot measurements of their peripheral oxygen saturation (SpO_2_), while in the background, heart rate is measured intermittently and physical activity (steps) measured continuously. This device analyzes the ECG signals, calculates the heart rate (robust mean over 30 seconds), and classifies the rhythm into one of the following categories: AF, Sinus Rhythm, Noise, Inconclusive^1^. Scanwatch uses three types of sensors: i) dry electrodes (ECG), ii) optical sensors (SpO_2_ and pulse rate), iii) accelerometer (physical activity). The accuracy of SpO_2_ measurement was assessed with a standardized protocol (ISO 80601-2-61:2017) during mild, moderate and severe hypoxias against gold-standard SaO_2_. The accuracy was good: 3% root mean square deviation (RMSE) (N = 14 patients and 275 blood samples) (22). Rhythm classification by the Scanwatch was compared to the diagnosis by cardiologists on simultaneously taken 12-lead ECGs (AF: n=100, normal sinus rhythm: n=113, other arrhythmia: n=45). 6.9% (18/262) were classified as Noise by the algorithm. Excluding patients with a reference diagnosis of other arrhythmias than AF or a noisy recording, the sensitivity for AF detection was 0.963 (95% CI lower boundary 0.894), with a specificity of 1.000 (95% CI limits of agreement 0.967) (23).

The BPM Connect^©^ is an oscillometric blood pressure monitor. Its accuracy and precision were assessed in the general population as well as in pregnant women, including those with pre-eclampsia (24–26). In the general population (N = 85), for validation criterion 1, the mean difference ± SD between the reference and device measured blood pressure (BP) values was 0.6 ± 5.3 mmHg for systolic blood pressure (SBP) and 2.1 ± 4.3 mmHg for diastolic blood pressure (DBP). For criterion 2, the SD of the mean BP differences between the test device and reference BP per subject was 4.2/3.6 mmHg (SBP/DBP) (27). In pregnant women, the mean differences between the mercury standard oscillograph and device BP values in pregnancy (n = 45) were −0.5 ± 5.7 mmHg for systolic BP (SBP) and −0.8 ± 3.8 mmHg for diastolic BP (DBP). In the subgroup of preeclamptic patients (n = 15), the mean differences were 0.14 ± 5.5 mmHg for SBP and 0.39 ± 3.7 mmHg for DBP (28).

Bodyscan^©^ is conceived as a health station. Besides measuring weight, it performs multifrequency segmental bioimpedance analysis and outputs the content of (mass and %) fat, visceral fat, lean tissues, muscles, total water, extra-cellular and intra-cellular water; it performs a 30 seconds 6-lead ECG (4 limb electrodes are placed, 2 on the glass plate and 2 on the handle), and calculates heart rate and classifies the rhythm into one of four categories (AF, Sinus Rhythm, Noise, Inconclusive^2^); it estimates the aortic Pulse Wave Velocity (PWV)^3^ and computes a Vascular Age^4^ and it assesses the sudomotor function, an early indicator of peripheral neuropathy^5^. The ECG classification by Bodyscan was compared to the diagnosis by cardiologists on simultaneously taken 12-lead ECGs (AF: n=82, normal sinus rhythm: n=131, other arrhythmia: n=12, uninterpretable: n=8). 8.4% of the recordings were classified as Noise by the algorithm. Excluding patients with a reference diagnosis of other arrhythmias than AF or an uninterpretable recording, the sensitivity for AF detection was 0.98 (95% CI lower bound 0.92), and the specificity was 1.0 (95% CI lower bound 0.98) [Personal Communication]. PWV was compared to the carotid-femoral PWV measured with a Sphygmocor by trained specialists in general population. The bias was 0.25 m·s^-1^ and the SE was 1.39 m·s^-1^. This agreement with Sphygmocor is “acceptable” according to the ARTERY classification (29).

Bodyscan’s measurement of the sudomotor (sweating) function was compared to the Sudoscan (Impeto Medical) (N=147). The sensitivity and specificity to classify with respect to (wrt) a neuropathy score of 70 (no neuropathy *vs* mild neuropathy) were 0.91 (95% CI lower bound 0.83) and 0.966 (95% CI lower bound 0.881) respectively, and the sensitivity and specificity to classify wrt a neuropathy score of 50 (mild *vs* severe neuropathy) were 0.915 (95% CI lower bound 0.796) and 0.990 (95% CI lower bound 0.946) [in preparation].

The Sleep Analyzer^©^ consists of an air inflated pad connected to a pressure sensor. The pressure signal is decomposed into three channels: movement, respiration, and heart beats. In addition, a microphone permits detection of snoring and snorting. The device provides data on sleep (time asleep, sleep efficiency, wake periods and sleep stages), pulse rate and respiration rate, and computes an apnea-hypopnea index (not available in the US). The Sleep Analyzer was included in a polysomnography (PSG) study involving 42 participants in a sleep laboratory. The respiratory rate had a bias of −0.06 breaths per minutes and 95% limits of agreement (LOA) of [−0.6; 0.5] breath per minute. The heart rate had a bias of −0.8 and a 95% LOA of [−4.3; 2.7] beats per minute (30). Patients suspected to have obstructive sleep apnea syndrome completed a night at a sleep clinic with a simultaneous PSG and recording with the Sleep Analyzer (N=180). The sensitivity, specificity, and area under the receiver operating characteristic curve (AUROC) at thresholds of apnea-hypopnea index ≥ 15 events/h were, respectively, Se_15 =_ 88.0%, Sp_15 =_ 88.6%, and AUROC_15 =_ 0.926. At the threshold of apnea-hypopnea index ≥ 30 events/h, results were Se_30 =_ 86.0%, Sp_30 =_ 91.2%, AUROC_30 =_ 0.954. The average total sleep time from PSG and the Withings Sleep Analyzers was 366.6 (61.2) and 392.4 (67.2) minutes, sleep efficiency was 82.5% (11.6) and 82.6% (11.6), and wake after sleep onset was 62.7 (48.0) and 45.2 (37.3) minutes, respectively (31).

### Advanced analytics: expert systems, threshold alarms

These devices are installed with WiFi or Bluetooth, and some are also available in a cellular version (Scanwatch, Sleep Analyzer, BPM Connect, scale Body Pro, Thermo, and a glucometer) ^6^ provided with Withings Remote Patient Monitoring (RPM^©^) solution. In this RPM solution, devices are pre-configured, shipped to the patient, and immediately available for use. After each measurement, the devices analyze the data and upload the results (e.g., SpO_2_, body composition) to Withings HDS servers. Data visualization can be reviewed by the patient using the Withings Health Mate application on a smartphone, or with a web interface. Health Care Professionals (HCP) can also visualize their patient’s data *via* the Withings web-based RPM interface. Withings RPM can be integrated to virtually any electronic health care report. Patient’s data confidentiality and security are ensured through an ISO 27001 certified hosting infrastructure and strictly following international general data protection regulation (i.e., GDPR). This is presented in **Figure 2**.

### Feedback and alarm sent to user and reference center

Patients and clinicians can consult their data in the Withings Health Mate^©^ application if not using the RPM solution application. In the RPM solution, patients are guided by a digital assistant (application on a smartphone), to maintain their adherence to monitoring on a daily basis. The digital assistant educates patients about the program and helps tracking, and a HIPAA compliant proactive phone support is provided to help patients who have not been able to activate their device. A setup confirmation is sent when devices are first installed. Finally, it reminds patients to take their readings, and shares tips on how to build a habit. The HCP-facing interface of the RPM is organized to display a complete history of the patients: all the health data from day one, alerts history, recent team notes, and current procedural technology (CPT) code completion history.

## Clinical potential of digital remote tools: the example of dialysis-dependent chronic kidney disease patients’ management, the case for Withings technology

**Figure 3:**
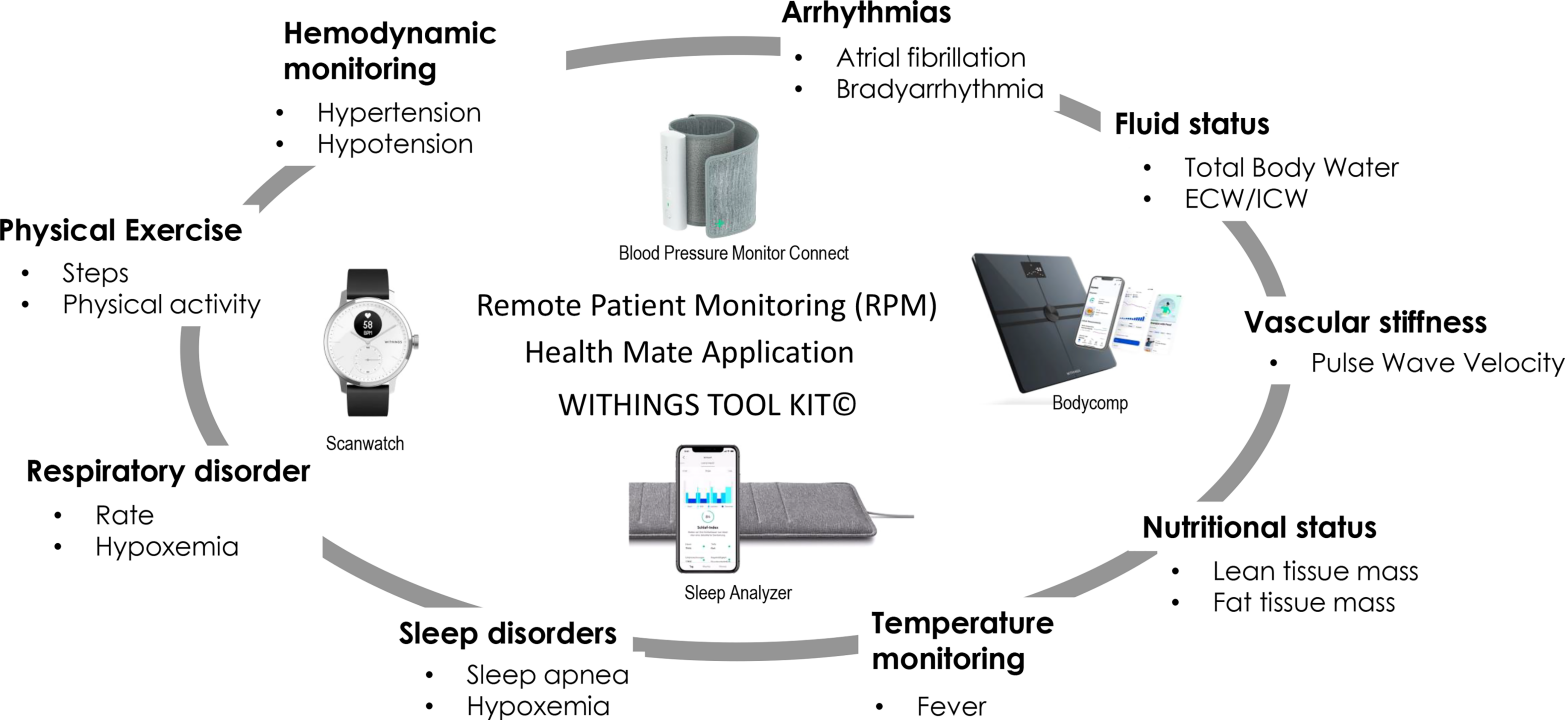
Expected results with support of digital remote monitoring.

Remote monitoring of changes in volume status, vital signs (blood pressure, heart rate, respiration rate, oxygen saturation), sleep quality and physical activity are among the most useful and valuable when assessing ambulatory CKD5D patients (32). Withings’ digital health toolkit devices offer a fully automated, unobtrusive and remote monitoring of the main vital functions in ambulatory subjects. These unique features provide a multidimensional assessment of ambulatory CKD5D patients covering most physiologic functions, detecting unexpected disorders (i.e., volume overload, arrhythmias) and finally helping physicians to judge patient’s response to treatment and recommendations. Among the various options provided with Withings’ digital health toolkit devices, we have briefly summarized some of the currently explored areas and potential fields for future clinical applications. Their potential use to support care of dialysis patients is presented in **Figure 3**.

Volume overload (VO) is a major cardiovascular risk factor for CKD5D patients. Chronic VO has been recognized as being a leading cause of hypertension and cardiac remodeling being independently associated with poor outcomes in dialysis patients (33–36). In addition, VO and pulmonary edema are leading causes of hospitalization (37, 38). Therefore, it seems crucial to carefully monitor volume status and blood pressure changes to avoid complications associated with volume overload. Volume changes may be easily assessed on a daily basis by means of a digital scale and/or multifrequency bioimpedance analyzer integrated into a digital scale. By combining this with daily blood pressure measurement given by the BPM Connect, it becomes possible to have an extensive hemodynamic assessment of CKD5D patients throughout the dialysis week. Permanent ambulatory respiratory rate and ECG measurements may also provide complementary information on patient volume overload and pulmonary edema risk. Chan et al. used a wearable patch biosensor to determine respiratory rates for remote patient monitoring and respiratory disease screening (39). For this purpose, they developed and validated an algorithm in a small population of 15 elderly subjects performing the different activities of daily living. Recognizing that volume overload contributes to reduced oxygenation, it can be anticipated that permanent monitoring of oxygen saturation (SpO_2_) may facilitate early detection and disease severity of volume overload. From a clinical perspective, by combining changes in bioimpedance scale, respiratory rate, oxygen saturation and ECG data generated by Withings’ toolkit, one can reasonably expect enhancing accuracy of detecting early volume overload in ambulatory CKD5D patients and to correct it before congestive heart failure develops, so avoiding acute hospital admission.

Arrhythmia is observed in 30 to 40% of established CKD5D patients leading potentially to life threatening complications including sudden death (40). It is well recognized that several pro-arrhythmic risk factors coexist in this population. However, the central abnormality that predisposes to arrhythmias is the cardiomyopathy, which is highly susceptible to abnormal ventricular conduction either spontaneously or *via* additional triggers such as dialysis-induced circulatory stress or electrolyte fluxes (41, 42). It has been also shown that some ECG abnormalities are prone to life-threatening arrhythmias that include heart-rate variability (HRV), ventricular late potentials, and QT interval prolongation. Using continuous ECG monitoring either with patch-based or implanted loop recording, it was shown that arrhythmias are more frequent than expected and ventricular tachycardia and/or tachyarrhythmia were the most frequent but clinically serious events were mainly observed with bradyarrhythmia’s (43). Roy-Chaudhury et al. have identified in a prospective study performed in 66 hemodialysis patients using an implantable loop recorder (ILR), that clinically serious arrhythmias (CSA) occurred in 67% of patients (44). Interestingly, arrhythmic disorders were distributed as follows: 77.3% had non-sustained ventricular arrhythmias, 20% had bradycardia, 9% had asystolic events and only one episode of sustained ventricular tachycardia was observed. Furthermore, it has been shown that arrhythmias were strongly related to the dialysis cycle. Prevalence of arrhythmia was highest during the first dialysis session of the week and also increased during the last 12 hours of each inter-dialytic interval and particularly with the longer interdialytic interval. In addition, electrolyte fluxes and dialysis prescription were associated with significant impacts on the incidence of arrhythmias. Sacher et al. provided further insights in a prospective study including 71 hemodialysis patients using an ILR device (19, 44). Twelve patients (17%) had a past history of AF or flutter. During a mean follow-up period of 21.3 months, 16 patients died (14% patient-years), 7 (44%) of cardiovascular causes and 4 sudden deaths occurred, with progressive bradycardia followed by asystole. *De novo* AF or flutter was diagnosed in 14 patients (20%). The incidence of patients presenting with clinically serious event and ventricular arrhythmia was 14% and 9% patient-years, respectively. In multivariate analyses, they identified several pro-arrhythmic risk factors that included high plasma potassium, low bicarbonate, high hemoglobin, high blood pressure, longer interdialytic period, history of coronary artery disease, previous arrhythmias and diabetes mellitus. Furthermore, specific risk associated with ventricular arrhythmia was low plasma potassium, no antiarrhythmic drugs and past history of arrhythmias (19, 44). Early detection of clinically serious arrhythmias potentially leading to cardiac arrest in high risk CKD5D patient is clearly an unmet medical need that requires more appropriate wearable and ultralight tools. Now, it remains to be proved in the clinical setting of hemodialysis that wireless patch device may offer comparable information to implantable wire loop recorders in an easier and more cost-effective manner (32, 42). From a clinical perspective, detection of clinically significant arrhythmias will lead to revision of renal replacement treatment options (i.e., intensive dialysis, dialysate electrolyte prescription) and referral to cardiology expertise to mitigate cardiac risk (i.e., medication, implantable pacemaker or defibrillator).

Sleep disorders are common and more frequent in CKD5 patients on maintenance hemodialysis than in the general population (45–47). In brief, among most frequently reported symptoms are insomnia (69%), obstructive sleep apnea syndrome (OSAS) (24%), restless leg syndrome (RLS) (18%) and excessive daytime sleepiness (EDS) (12%). Furthermore, sleep disorders are strongly associated with poor health-related quality of life (i.e., fatigue) and poor patient outcomes (i.e., higher cardiac burden) (48, 49). Several risk factors have been identified including non-modifiable factors (e.g., obesity, diabetes, chronic kidney disease) and modifiable factors in particular dialysis adequacy (e.g., volume overload, hypertension, anemia, albumin). Automated prediction of apnea-hypopnea index and detection of sleep apnea is an interesting approach to reduce a newly identified cardiovascular risk factor in CKD5D population. Apnea-Hypopnea Index (AHI) assesses severity of this disorder by measuring number of apnea or hypopnea events per hour. Usually, between 2% and 5% of adult women and 3% to 7% of adult men have sleep apnea syndrome (SAPS). Selvaraj et al. have used a patch biosensor to estimate the apnea-hypopnea index in a cohort of high-risk subjects (50). For this purpose, they developed an algorithm using features provided by statistical and filtering dispersion analysis of the nasal airflow respiration signals. Based on this algorithm, they computed apnea or hypopnea occurrences on a per-second basis and compared the predictive value of their algorithm to the ‘gold standard’ relying on a polysomnography (PSG) record. Interestingly, they showed that the predictive value of the patch biosensor was comparable to the apnea-hypopnea index obtained with the PSG. The authors confirmed that patch biosensor can be used in sleep apnea syndrome screening as an inexpensive and convenient solution for recurrent sleep apnea evaluation. In addition, it has been shown recently that about 10% of hemodialysis patients presented with prolonged and relatively severe hypoxemia during dialysis sessions. Hypoxemic patients have higher mortality risk estimated around 20% at one year (51, 52). Now, it remains to be shown that wearable wireless bed patch biosensors (sleep analyzer) have the same accuracy in CKD5D patients. From a clinical perspective, patients presenting with high apnea-hypopnea index and/or prolonged hypoxia may benefit from intermittent continuous positive airway pressure (CPAP) devices and/or oxygen therapy to mitigate this cardiac risk.

Impaired physical activity is a key feature of adults with advanced CKD, and is associated with poor outcome and increased mortality (53, 54). The physical fitness in aged dialysis patients is so reduced that it impinges on ability to perform everyday activities of life and domestic tasks and increases the risk of falls. An increasing number of studies have stressed health benefits of regular exercise training programs to the point that they are perceived as a cornerstone of therapy (55–58). Monitoring physical activity and guiding physical exercise training in an adapted and structured way are of crucial importance in this frail population. In this context, wearable wireless connected patch sensors provide monitoring to achieve this goal in an easy and transparent way. Using a wireless patch sensor, Selvaraj et al. have assessed and confirmed performances and accuracy of such device in measuring vital signs (59). In this study, 76 senior participants were included and were followed up to 3603 days. The results confirmed not only wearability and usability of the wireless biosensor at home over the longer term but also showed the clinical value in the early detection of falls. Usually, experiments target young participants and some activities of daily living in assessing the specificity and sensitivity of fall detection systems. The Selvaraj study has also shown the suitability of wireless patch sensor in elderly participants for long-term monitoring at home. In another study, Chan et al. compared accuracy of a wireless patch sensor with more conventional medical devices (39). In brief, they confirmed that patch connected biosensor provides similar results than larger and traditional tools in measuring physical activity and vital signs.

Protein energy wasting (PEW) is more common in advanced CKD and dialysis patients (20-50%) and also associated with an increased risk of morbidity and mortality (60, 61). Early recognition and treatment of malnutrition are essential to improve the outcome of CKD and dialysis patients. Assessment of PEW relies on a series of markers including clinical and anthropometric signs (weight loss, low body mass index, lean tissue mass), laboratory investigations (albumin, transthyretin), dietary survey (protein and energy intake) and instrument tools (bioimpedance, energy expenditure) (62, 63). Diagnosis of PEW is usually recognized by health care professionals as cumbersome or made at a too late stage. In this context, wearable patch wireless sensor devices might offer a new and convenient tool for an early detection of reduced daily energy expenditure and physical activity. Furthermore, the combined use with body composition analyzer using bioimpedance assessments will track lean tissue mass wasting. Selvaraj et al. have used a wireless patch biosensor for assessing energy expenditure (EE) rate and total daily energy expenditure (TEE) in a cohort of 32 ambulatory subjects aged 21 to 72 years old with a wide range of body mass index (64). Their results confirmed that patch biosensors allow accurate measurement of EE and TEE and provide a reliable and non-constraining tool for such assessments. Now, it remains to be proved that accuracy of such wearable patch devices equally works in CKD5D patients, that requires further validation studies.

Falls among aged CKD5D patients are a serious concern since they have significant impact on quality of life and mortality (65). Falls are also a significant cause of morbidity that contributes to a substantial burden to health care costs. Although falls may be predicted or minimized, they are not totally preventable. Fall reduction in elderly CKD5D patients is an important focus for health care researcher (66, 67). Narasimhan et al. used a biosensor to automatically detect human falls (68). The tri-axial accelerometer, Bluetooth Low Energy transceiver, and microcontroller found in the sensor can detect a possible fall if there is impact and the person lies horizontally after the fall. Furthermore, the activity after the fall must be below the threshold. The study recruited 15 elderly volunteers to perform activities of daily living and 10 volunteers to perform intentional falls using a gymnastics mat. The algorithm developed offered 100% specificity and 99% sensitivity. From a clinical perspective, digital remote self-operated tools may provide an easy means to monitor physical activity and energy expenditure on one hand, and to activate patients through personalized coaching and training through advice on the other.

## Challenges and threats of digital health technology

The introduction of a new technology, particularly in patient care, is always subject to caution, fears or even doubts by medical community experts. This is the case for digital health systems and particularly for pervasive remote monitoring devices that bring another level of complexity to patient management. In this paragraph, we provide few thoughts about challenges and threats related to the intrusion of digital health technology in tomorrow medical field.

On one end, there are some challenges that deserve to be highlighted: Firstly, amount of big data generated by these devices when used on a daily basis; Secondly, usability of this information by users and care givers; Thirdly, reliability of medical information generated and transmitted to care professionals; Fourthly, safety of patients’ data protection system; Fifthly, integration of these tools in standard of care and patient workflow.

On the other end, there are also threats that need to be underlined: Firstly, reliability of information captured and transmitted to care givers; Secondly, data interpretation and translation in an active patient management plan; Thirdly, patient burned out associated with continuous monitoring perceived as too intrusive; Fourthly, economical value based of this remote digital health technology must be assessed more precisely; Fifthly, excessive reliance on digital health system may lead to loss of medical expertise or doctor/patient interaction, this is a risk.

All these challenges and threats must be kept in mind since they will deserve more attention and evaluation in the future. It is not our purpose to elaborate further on these topics, due to space restriction, but also because we are at the dawn of new technological revolution in the health care system, and answers will be provided with more extensive use of these digital health tools.

## Expected results and future development

Increasing advances in technology, especially electronics and computerization, will lead to a new generation and wider availability of such pervasive health sensing and digital technology at an acceptable cost, that will allow extensive non-invasive monitoring of fragile CKD5D patients at home. As discussed earlier, regular monitoring of vital functions may help care givers to improve the management of dialysis patients by providing early alert to serious events such as volume overload and/or arrhythmias before life-threatening complications occur.

By combining remote extensive multiplexed sensing technology and advanced analytics relying on cloud computing and artificial intelligence, and RPM management, Withings’ tool kit potentially offers today a convenient and cost competitive means of improving care of CKD patients. Newer generations of this highly sophisticated technology should provide a major step towards precise and personalized medicine. It has potential to improve patient perception, to reduce cardiovascular burden and finally to reduce costs associated with hospitalization.

In the future, it remains to be determined how these tools work for CKD patients and which category of patients will benefit more from this wearable sensing and digital technology. Further studies are needed to identify potential clinical benefits and to position these tools in the clinical management of CKD patients, and in particular in the renal replacement therapy field.

## Take home message

Digital self-operated tools such as Withings’s toolkit provide a new and integrated approach for monitoring ambulatory dialysis patients. A number of these devices are already commercially available and potentially offer a fully automated, unobtrusive and remote monitoring of main vital functions in ambulatory subjects. It was not our intent to compare accuracy and usability of these digital health tools, but rather to use case of on one comprehensive and integrated digital health toolkit system in improving care of CKD patients.

These unique features provide a multidimensional way of assessing ambulatory CKD5D patients covering most of the relevant physiologic functionalities, detecting unexpected disorders (i.e., volume overload, arrhythmias, sleep disorders), providing alarms to patients and supervising clinical center and finally supporting physicians to judge patient responses to treatment and recommendations. Although this new technology has the potential to improve the quality of care for CKD5D patients, further studies are required to confirm the prospective clinical benefits of this rapidly developing technology.

## Author contributions

All authors contributed to the article and approved the submitted version.

## Footnotes

1 ‘Noise’ is an output given if the quality of the recording is too poor to classify the rhythm with confidence. ‘Inconclusive’ is the output when the rhythm cannot be classified as either AF or Sinus Rhythm with enough confidence. This may happen when the heart rate is < 50 bpm or > 150 bpm, or in the presence of another arrhythmia than AF.

2 Cf footnote 1.

3 The ballistocardiogram, recorded as small variations of weight produced by the recoil of the center of mass upon the ejection of blood into the aorta, gives a proximal time point. An impedance plethysmogram of the foot (measuring the variations of conductivity) gives a distal time point. A model using these time points and the height of the patient was trained to output the carotid-femoral PWV measured with a Sphigmocor (AtCor Medical).

4 The Vascular Age is the chronological age of the patient corrected by a PWV-dependent factor.

5 It combines reverse iontophoresis, i.e., migration of some ions from the human skin to the electrodes in contact with the sole of the feet, and steady multiple chronovoltammetry, which is the application of rectangular direct current (DC) voltages of varying amplitudes. A time/ampere curve is recorded. Electrochemical skin conductance (ESC) in the hands and feet, i. e., the ratio between current generated and the constant DC voltage stimulus, is calculated and compared to normal values.

6 Availability of the cellular devices depends on the geographical area. The RPM application is available in Europe but not in the US.
